# Development of genome-specific primers for homoeologous genes in allopolyploid species: the *waxy *and *starch synthase II *genes in allohexaploid wheat (*Triticum aestivum *L.) as examples

**DOI:** 10.1186/1756-0500-3-140

**Published:** 2010-05-24

**Authors:** Xiu-Qiang Huang, Anita Brûlé-Babel

**Affiliations:** 1 Department of Plant Science, University of Manitoba, 66 Dafoe Road, Winnipeg, Manitoba R3T 2N2, Canada

## Abstract

**Background:**

In allopolypoid crops, homoeologous genes in different genomes exhibit a very high sequence similarity, especially in the coding regions of genes. This makes it difficult to design genome-specific primers to amplify individual genes from different genomes. Development of genome-specific primers for agronomically important genes in allopolypoid crops is very important and useful not only for the study of sequence diversity and association mapping of genes in natural populations, but also for the development of gene-based functional markers for marker-assisted breeding. Here we report on a useful approach for the development of genome-specific primers in allohexaploid wheat.

**Findings:**

In the present study, three genome-specific primer sets for the *waxy *(*Wx*) genes and four genome-specific primer sets for the *starch synthase II *(*SSII*) genes were developed mainly from single nucleotide polymorphisms (SNPs) and/or insertions or deletions (Indels) in introns and intron-exon junctions. The size of a single PCR product ranged from 750 bp to 1657 bp. The total length of amplified PCR products by these genome-specific primer sets accounted for 72.6%-87.0% of the *Wx *genes and 59.5%-61.6% of the *SSII *genes. Five genome-specific primer sets for the *Wx *genes (one for Wx-7A, three for Wx-4A and one for Wx-7D) could distinguish the wild type wheat and partial waxy wheat lines. These genome-specific primer sets for the *Wx *and *SSII *genes produced amplifications in hexaploid wheat, cultivated durum wheat, and *Aegilops tauschii *accessions, but failed to generate amplification in the majority of wild diploid and tetraploid accessions.

**Conclusions:**

For the first time, we report on the development of genome-specific primers from three homoeologous *Wx *and *SSII *genes covering the majority of the genes in allohexaploid wheat. These genome-specific primers are being used for the study of sequence diversity and association mapping of the three homoeologous *Wx *and *SSII *genes in natural populations of both hexaploid wheat and cultivated tetraploid wheat. The strategies used in this paper can be used to develop genome-specific primers for homoeologous genes in any allopolypoid species. They may be also suitable for (i) the development of gene-specific primers for duplicated paralogous genes in any diploid species, and (ii) the development of allele-specific primers at the same gene locus.

## Background

Polyploidy is recognized as a major force in plant speciation, with approximately 70% of flowering plants being polyploid [1]. Polyploids are divided into autopolyploids and allopolypoids. Allopolyploids contain two or more sets of related chromosomes that are brought together into the same nucleus by interspecific hybridization and followed by spontaneous chromosome doubling or unreduced gametes in nature [2]. Allopolyploids are preponderant in nature and include the world's most important crops such as cotton, canola, oat and wheat. In allopolypoid crops, homoeologous genes in different genomes exhibit a very high sequence similarity, especially in the coding regions of genes [3,4]. This makes it difficult to design genome-specific primers to amplify individual genes from different genomes for sequence diversity and association mapping. Development of genome-specific primers for agronomically important genes in allopolypoid crops will facilitate not only the study of sequence diversity and association mapping of genes in natural populations, but also the development of gene-based functional markers for marker-assisted breeding. Here we describe a useful approach for the development of genome-specific primers in allopolyploid species.

Bread wheat (*Triticum aestivum *L.) is an allohexaploid species, consisting of three sets of subgenomes (A, B, and D), each of which contains seven pairs of homoeologous chromosomes. Its grain is the staple food for 35% of the world's population http://www.cimmyt.org/. The biosynthesis of starch is the major determinant of yield in wheat grains, because starch accounts for about 70% of grain dry weight [5]. Starch is composed of approximately 75% amylopectin and 25% amylose. Amylose is a relatively less-branched α-(1→4)-linked glucose polymer, whereas amylopectin is a branched glucose polymer in which α-(1→4)-linked polymer are connected by α-(1→6)-linkages. Starch synthesis involves a series of biosynthetic enzymes including ADP-glucose pyrophosphorylase (AGP), starch synthases (SS) and starch branching enzymes (SBE). Two distinct types of starch synthases, isoforms of granule-bound starch synthase (GBSSI, also known as Waxy) and soluble starch synthases (SSI, SSII and SSIII), are involved in the conversion of ADP-glucose to the starch polymers. The GBSSI is the major enzyme responsible for amylose production, whereas the soluble starch synthases with starch branching enzymes are thought to be involved in amylopectin synthesis [6]. Starch synthase II is thought to be involved in elongating the short chains of amylopectin [6]. SSII has a gene dosage effect on grain weight in wheat and the seed weight of the *SSII *triple null lines decreased significantly compared to the other *SSII *genotypes [7]. The starch of the mutant lacking GBSSI is amylose-free, while the mutant lacking SSII contains a high level of amylose in the starch [8,9]. The seed of the *GBSSI*/*SSII *double null mutant was severely shrunken and the seed weight reduced [10].

It is known that three homoeologous *SSI *and *SSIII *genes are located on group 7 chromosomes and group 1 chromosomes, respectively [11,12]. Three Waxy (*Wx*) loci, *Wx-A1, Wx-B1 *and *Wx-D1*, are located on chromosome arms 7AS, 4AL (translocated from 7BS) and 7DS [13,14], while three homoeologous *SSII *genes, *SSII-A*, -*B *and -*D*, coding SGP (starch granule protein-1) -A1, -B1, and -D1, are located on chromosome arms 7AS, 7BS and 7DS [12,15], respectively. To date, three homoeologous genomic sequences are available for the *Wx *and *SSII *genes [13,15]. Only cDNA sequences or genomic sequences of *Aegilops tauschii*, the donor of the D-genome of hexaploid wheat, have been isolated and cloned for the other genes involved in starch synthesis [12].

The sequence diversity and association mapping of starch biosynthesis genes has been conducted in diploid cereal crops such as barley [16-18], maize [19,20], rice [21,22], sorghum [23] and foxtail millet [24]. The allohexaploid nature of common wheat and the homoeologous genes from the genomes A, B and D with a high level of sequence similarities in the coding regions make it very difficult to design genome-specific primers [3,4]. Development of one primer set from any region of gene in each genome based on large InDels (insertions or deletions) is not difficult. However, it is more challenging to develop many genome-specific primer sets from the homoeologous sequence regions, which cover the entire genes. Blake et al. [25] developed genome-specific primers for the *Wx *genes, however, only one primer set was designed for each genome and three genome-specific primers were not developed from the homoeologous sequence regions. Genome-specific primers for the *SSII *genes have not been reported so far. The objectives of the present study were to (i) develop genome-specific primers from the homoeologous sequence regions of both the *Wx *and *SSII *genes, (ii) determine whether genome-specific primers for the *Wx *genes were able to distinguish the wild type wheat and partial waxy wheat lines, and (iii) determine whether genome-specific primers were able to amplify the *Wx *and *SSII *genes in its progenitors, tetraploid wheat lines and diploid species.

## Results and Discussion

### Sequence similarities of the *Wx *and *SSII *genes

Genomic sequences of the *Wx *and *SSII *genes have been isolated by Murai et al. [13] and Shimbata et al. [15], respectively. Three homoeologous *Wx *genes consist of 11 exons and 10 introns. The length of their genomic sequences ranges from 2781 bp for *Wx-7A *(AB019622) to 2862 bp for *Wx-7D *(AB019624). Three homoeologous *SSII *genes contain 8 exons and 7 introns. The length of their genomic sequences ranges from 6566 bp for *SSII-7B *(AB201446) to 6775 bp for *SSII-7D *(AB201447). The coding regions (CDs) of three *Wx *genes range in length from 1815 bp to 1818 bp, while the length of the CDs of three *SSII *genes varies from 2397 to 2400 bp. Sequence alignment indicated that sequence similarities of the CDs were more than 95.0% among the three homoeologous *Wx *genes and over 96.0% among the three homoeologous *SSII *genes. Therefore, it is very difficult to design more genome-specific primer sets from the CDs to cover the *Wx *and *SSII *genes.

**Figure 1:**
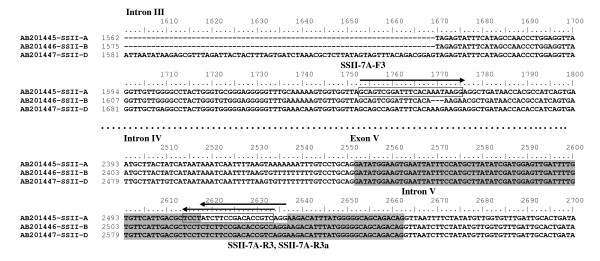
**Alignment of partial genomic DNA sequences of three homoeologous *SSII *genes from hexaploid wheat indicating the position of genome-specific primers**. The exon and intron regions are shown with gray and white color, respectively. Deletions are indicated by *dashes*. The sequence of the forward primer and complementary sequences of the reverse primers are *boxed*.

In general, more genetic variations occur in intron regions than in exon regions in grass genomes [26]. Sequence alignment of the intron regions showed that intron sequence similarities ranged from 68.9% to 74.4% among the three homoeologous *Wx *genes and from 68.7% to 82.1% among the three homoeologous *SSII *genes. Many SNPs (single nucleotide polymorphisms) and/or InDels (insertions or deletions) were detected in the intron regions (Figure 1). This provides strategies to design genome-specific primer sets from the SNPs and/or Indels of the intron regions.

### Development of genome-specific primers for the *Wx *and *SSII *genes

Genome-specific primers are required to investigate nucleotide diversity of the three homoeologous *Wx *and *SSII *genes and to conduct association analysis between the three homoeologous *Wx *and *SSII *genes and grain yield-related traits. To be useful, the primers should be developed to meet the following criteria: (1) the amplified fragment must be genome-specific; (2) the fragment length should be from 800 bp to 1500 bp for the complete sequencing using both forward and reverse primers by means of ABI 3730 × l DNA Analyzer (Applied Biosystems, CA); (3) the neighboring amplicons should overlap by 80 bp to 100 bp, so that multiple sequences of two or more amplicons can be assembled together after direct sequencing of the amplicons.

Multiple mismatches especially a mismatch of the 3' terminus of a primer between one genome sequence and sequences of other two genomes were required to obtain the genome specificity of the primer. After sequence alignment, both forward and reverse primers were manually designed by picking primers ending with a mismatch (SNP) at the 3' terminus. Primer3 http://frodo.wi.mit.edu/primer3/ were used to check general parameters of manually picked primers. Melting temperature (Tm) of both primers ranging from 59°C to 61°C was adjusted by adding or deleting nucleotides at the 5' terminus of primers. Primer length from 17 nt to 25 nt worked well. Other parameters were used as default. Annealing temperature 60°C was used for PCR.

**Figure 2:**
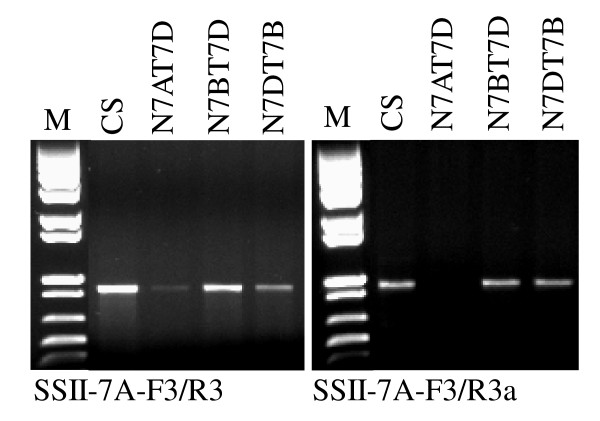
**A comparison between unspecific (SSII-7A-F3/SSII-7A-R3) and specific (SSII-7A-F3/SSII-7A-R3a) primer sets**. Each primer set was used to amplify Chinese Spring (CS), N7AT7D, N7BT7D and N7DT7B. M = 1 kb plus DNA ladder.

Only one genome-specific primer based on one mismatch at the 3' terminus in a primer set and both genome-specific forward and reverse primers based on mismatch(s) in the middle or beginning of primers did not guarantee genome-specific amplification. In the absence of a perfect match between a primer and a template, primer-template mismatch amplification may be generated in the first cycle of PCR (e.g. nulli-tetrasomic lines). Huang and Cloutier [3] used BAC DNA as template and primer-template mismatch PCR to identify a number of genes for low molecular weight glutenin subunits (LMW-GS) in hexaploid wheat. In Figure 1, when a SSII-7A gene-specific forward primer SSII-7A-F3 from Intron III combined with a reverse primer SSII-7A-R3 from Exon V with one mismatch T/C at the fifth position from the 3'-terminus of the primer, a weak fragment appeared in N7AT7D (Figure 2). This fragment was most likely amplified from the SSII gene on chromosomes 7D by primer-template mismatch PCR. When the specific forward primer SSII-7A-F3 combined with a specific reverse primer SSII-7A-R3a from Exon V with one mismatch T/C at the 3'-terminus of the primer, a specific fragment was produced from chromosome 7A (Figures 1 and 2).

**Figure 3:**
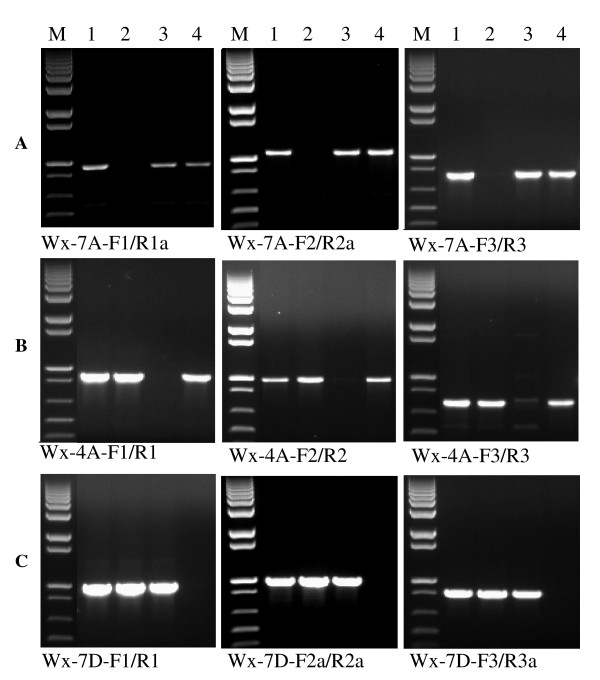
**Genome-specific PCR amplification for the three homoeologous *Wx *loci**. Each primer set was used to amplify Chinese Spring (CS, lane 1), N7AT7D (lane 2) and N4AT4D (lane 3) and N7DT7B (lane 4). M = 1 kb plus DNA ladder. PCR amplification with (A) 7A-specific primer sets, (B) 4A (B genome)-specific primer sets, and (C) 7D-specific primer sets.

All primer sequences and amplicon information for the three homoeologous *Wx *and *SSII *genes are listed in Table 1. For the three homoeologous *Wx *genes, three genome-specific primer sets for all three genomes were developed (Table 1 and Figure 3). The forward primers of primer set 1 were located in Intron I (Wx-7A-F1 and Wx-4A-F1) or at Exon I/Intron I boundary (Wx-7D-F1), while the reverse primers were designed from Intron V (Wx-4A-R1 and Wx-7D-R1) or Intron V/Exon VI junction (Wx-7A-R1a). The forward primers of primer set 2 were developed from Intron V (Wx-7A-F2), Intron IV (Wx-4A-F2), and Exon V (Wx-7D-F2a), respectively. The reverse primers of primer set 2 were located in Intron VIII (Wx-7A-R2a), Intron VII (Wx-4A-R2), and Exon VIII (Wx-7D-R2a), respectively. The forward primers of primer set 3 were developed from Intron VI (Wx-4A-F3) or Exon VII/Intron VII junction (Wx-7A-F3 and Wx-7D-F3), whereas the reverse primers of primer set 3 were located in Intron X (Wx-7A-R3), Intron IX (Wx-4A-R3), and Exon XI (Wx-7D-R3a), respectively. The size of a single PCR product varies from 731 bp to 1038 bp (Figure 3). The genome specificity of these nine primer sets were determined through amplifying genomic DNA of Chinese Spring (CS), N7AT7D, N4AT4D (the Wx-B1 locus was assigned to a segment of chromosome 7B translocated to chromosome 4A) [13], and N7DT7B. The 7A-specific primer sets amplify a single PCR product from CS, N4AT4D and N7DT7B, but not from N7AT7D. The amplicons produced by the 4A (B genome)-specific primer sets were absent in N4AT4D, but present in CS, N7AT7D and N7DT7B. The 7D-specific primer sets generated a single PCR product from CS, N7AT7D and N4AT4D, but not from N7DT7B (Figure 3). The total length of PCR products amplified by the three primer sets accounted for 72.6% of the *Wx-4A *gene, 82.9% of the *Wx-7A *gene and 87.0% of the *Wx-7D *gene.

**Table 1:** Genome-specific primer sets for the three homoeologous *Wx *and *SSII *genes.

Primer name	Primer sequence (5'→3')	Tm (°C)	Size (bp)	Chromosomal location
Wx-7A-F1	GTAAGCTTGCGCCACTGC			
Wx-7A-R1a	GGATGCAGAATGCCACCTA	60	950	7A
Wx-7A-F2	CGCTCTGCATATCAATTTTGC			
Wx-7A-R2a	ATATGCAAAGGAGGTGAGGAAC	60	1038	7A
Wx-7A-F3	CTGGTACGATCGACCGACAT			
Wx-7A-R3	CGGCCCTTCACTCTTAGTTG	60	750	7A
Wx-4A-F1	AGCTAGCACCACTGTCTTCTGA			
Wx-4A-R1	GGCCGTCCTATAGATGCCAC	60	854	4A
Wx-4A-F2	TCAACAACACCCAGCAGCTA			
Wx-4A-R2	GGTTGGGGTCGATGACGTA	60	943	4A
Wx-4A-F3	CCACACACCCACACAAAGAT			
Wx-4A-R3	TTTACACAAGGGATCGACGAG	60	731	4A
Wx-7D-F1	CCATGGCCGTAAGCTAGAC			
Wx-7D-R1	CGCAAAATTGATATGCCTGTT	60	978	7D
Wx-7D-F2a	AACTACCAGTCCAATGGCATCTAC			
Wx-7D-R2a	GCTCGGGAATTTCTCCTCAAT	60	938	7D
Wx-7D-F3	CCAGATCGTTCTCCTGGTACA			
Wx-7D-R3a	CTCGCTCCCCTCGACA	60	861	7D
SSII-7A-F2	CCCAGAACAGAGTACCAGTGAAC			
SSII-7A-R2	CGGATCTACAGGGCAGGTAA	60	953	7A
SSII-7A-F3	GCAGTCGGATTTCACAAATAAGG			
SSII-7A-R3a	CCTGACGGTGTCGGAAGAT	60	883	7A
SSII-7A-F4a	AGCGAAAATGCAATCAAAGG			
SSII-7A-R4a	TTTGGGTATGAGGGGGAAAT	60	1657	7A
SSII-7A-F5a	TGCACCATCGCTCGAAGT			
SSII-7A-R5	CGTTGATGTGACACCATATCCT	60	983	7A
SSII-7B-F2	CTGTCAGCGACGTGGAACT			
SSII-7B-R2	TGCATTGAAATGAAAGCTTGAC	60	947	7B
SSII-7B-F3a	GCAGTCGGATTTCACAAAGAAC			
SSII-7B-R3	GGTCAGTAGGCCTTGGCTTG	60	973	7B
SSII-7B-F4a	CATTGACGCTCCTCTCTTCC			
SSII-7B-R4a	TACTCCCACTATGGTTAGCCTTACA	60	1596	7B
SSII-7B-F5	ACAACTTCATGGGAACAAGGTT			
SSII-7B-R5	CTCAGACCTGACGGAGATGG	60	949	7B
SSII-7D-F2a	GTCAGCGACGTGGAACAA			
SSII-7D-R2a	CGCGAAACTAGCTCCCAATC	60	1053	7D
SSII-7D-F3	AGCCAGATTTCACAAAGAAGGA			
SSII-7D-R3	AGTCAGTAGGCCTTGGCTTG	60	973	7D
SSII-7D-F4	CTCTCTTCCGACACCGTCA			
SSII-7D-R4	GGAGAAGGAGAGGAGAAGTTGG	60	1654	7D
SSII-7D-F5	TGCGTCGCCTCATAGAGC			
SSII-7D-R5	GCACAAGCAACTGACCTCAC	60	987	7D

The forward and reverse primer pairs are listed together for each fragment amplified.

**Figure 4:**
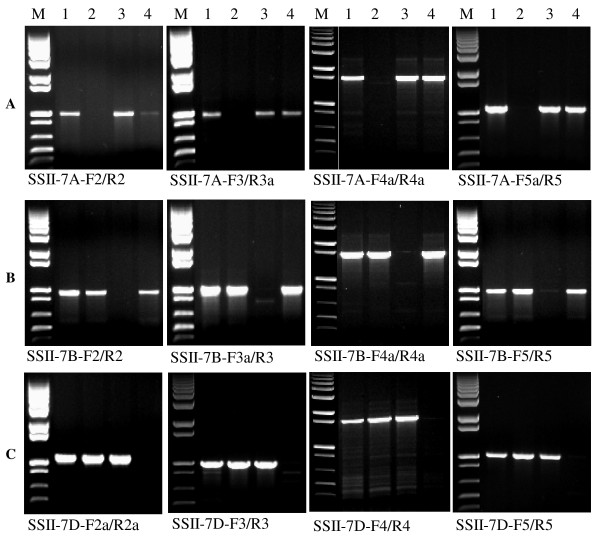
**Genome-specific PCR amplification for the three homoeologous *SSII *loci**. Each primer set was used to amplify Chinese Spring (CS, lane 1), N7AT7D (lane 2) and N7BT7D (lane 3) and N7DT7B (lane 4). M = 1 kb plus DNA ladder. PCR amplification with (A) 7A-specific primer sets, (B) 7B-specific primer sets, and (C) 7D-specific primer sets.

For the three homoeologous *SSII *genes, four genome-specific primer sets for all three genomes were designed (Table 1 and Figure 4). The forward primers of primer set 2 were located in Exon II, while the reverse primers were designed from Intron III. The forward primers of primer set 3 were developed from Intron III. The reverse primers of primer set 3 were located in Exon V (SSII-7A-R3a) and Intron V (SSII-7B-R3 and SSII-7D-R3), respectively. The forward primers of primer set 4 were developed from Intron IV (SSII-7A-F4a) or Exon V (SSII-7B-F4a and SSII-7D-F4), whereas the reverse primers were located in Intron VII. Both the forward and reverse primers of primer set 5 were designed from Intron VII. The size of a single PCR product ranges from 883 bp to 1657 bp. The genome specificity of these four primer sets were confirmed through amplifying genomic DNA of CS, N7AT7D, N7BT7D and N7DT7B (Figure 4). The total length of amplified PCR products by the four primer sets covered 59.5% of the *SSII-7B *gene, 60.3% of the *SSII-7D *gene and 61.6% of the *SSII-7A *gene.

These genome-specific primer sets for the three homoeologous *Wx *and *SSII *genes were used to amplify 90 bread wheat genotypes, which were originated from Canada, USA, UK and Germany. Single PCR products were recovered in the all 90 wheat genotypes by using each of the genome-specific primer sets (data not shown). The result indicated that the regions used to design the primers were conserved and no genetic variation occurred in these regions during wheat domestication and artificial selection in wheat breeding. These genome-specific primers are being used for the study of sequence diversity and association mapping of the three homoeologous *Wx *and *SSII *genes in natural populations of hexaploid wheat.

### Distinguishing the wild type wheat and partial waxy wheat lines using genome-specific primers for the *Wx *genes

**Figure 5:**
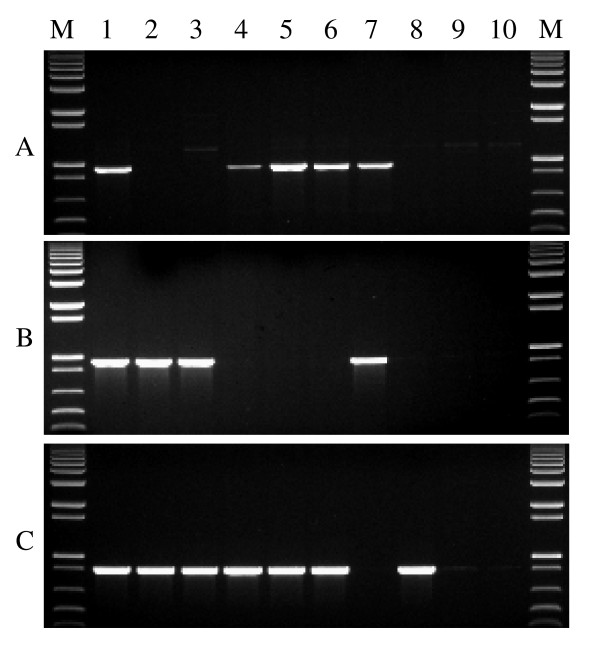
**PCR amplification for distinguishing null alleles of the *waxy *genes and wild type in wheat**. (A) PCR results with primer set Wx-7A-F1 and Wx-7A-R1a to distinguish the wild type (*Wx-A1a*) and null allele (*Wx-A1b*) of the *Wx-A1 *gene. (B) PCR results with primer set Wx-7B-F2 and Wx-7B-R2 to distinguish the wild type (*Wx-B1a*) and null allele (*Wx-B1b*) of the *Wx-B1 *gene. (C) PCR results with primer set Wx-7D-F3 and Wx-7D-R3a to distinguish the wild type (*Wx-D1a*) and null allele (*Wx-D1b*) of the *Wx-D1 *gene. M = 1 kb plus DNA ladder. Lanes: 1, CS; 2, Sturdy; 3, Fujimi Komugi; 4, Gabo; 5, Gamenya; 6, Santanta; 7, Bai Huo; 8, Kanto 107; 9, NSGC 8645; 10, NSGC 8646.

It has been reported that wheat varieties Sturdy and Fujimi Komugi carry the null *Wx-A1 *allele (*Wx-A1b*) [27-29] (Table 2). Gabo, Gamenya and Santanta possess the null *Wx-B1 *allele (*Wx-B1b*). The Chinese wheat variety Bai Huo has the null allele (*Wx-D1b*) at the *Wx-D1 *locus, whereas the Japanese wheat variety Kanto 107 carries the double null alleles *Wx-A1b *and *Wx-B1b *[30]. Morris and Konzak [31] developed soft and hard homozygous waxy wheat lines, NSGC 8645 (PI 612545) and NSGC 8646 (PI 612546), which both carry the triple null alleles *Wx-A1b*, *Wx-B1b *and *Wx-D1b*. The nine genome-specific primer sets for the three homoeologous *Wx *genes were used to amplify these partial waxy wheat genotypes and breeding lines. It was found that one out of three 7A-specific primer sets, Wx-7A-F1/Wx-7A-R1a, could distinguish the wild type allele *Wx-A1a *in CS (PCR product present) and the null allele *Wx-A1b *in Sturdy, Fujimi Komugi, Kanto 107, NSGC 8645 and NSGC 8646 (absent) (Table 2 and Figure 5). Further comparison of aligned sequences from *Wx-A1a *and *Wx-A1b *indicated that the forward primer Wx-7A-F1 was designed from the beginning of Intron I, which was deleted in the sequence of the null allele *Wx-A1b *[14]. The PCR product of three 4A (B genome)-specific primer sets was present in the wild type allele *Wx-B1a *in CS, but absent in the null allele *Wx-B1b *in Gabo, Gamenya, Santanta, Kanto 107, NSGC 8645 and NSGC 8646. The PCR analysis of the primer set Wx-4A-F2/Wx-4A-R2 is shown in Table 2 and Figure 5. This agrees with the explanation that the entire coding region of the *Wx-B1 *gene was deleted in the null allele *Wx-B1b *[14,32]. One of three 7D-specific primer sets, Wx-7D-F3/Wx-7D-R3a, could be used to differentiate the wild type allele *Wx-D1a *in CS (PCR product present) and the null allele *Wx-D1b *in Bai Huo, NSGC 8645 and NSGC 8646 (absent) (Table 2 and Figure 5), because the reverse primer Wx-7D-R3a was designed from Exon XI, which was deleted in the sequence of the null allele *Wx-D1b *[14]. These results show that the genome-specific primer sets would have limited application to detect the null alleles for marker-assisted selection in the wheat breeding programs because of the absence of PCR products in the partial waxy wheat lines. Co-dominant markers have been developed for the selection of all the three null alleles at the *Wx *loci [27,32]. However, the primers for the most co-dominant markers were not genome-specific.

**Table 2:** Classification of the partial waxy wheat lines according to the presence (+) or absence (-) of each waxy protein and amplification results of each genome-specific primer set.

Genotype	Accession number	Country	Null allele	Type*	Wx protein			Genome-specific primer set		
						
					Wx-A1	Wx-B1	Wx-D1	Wx-7A-F1R1a	Wx-4A-F2R2	Wx-7D-F3R3a
Chinese Spring	-	China	Wild type	1	+ ^†^	+	+	+	+	+
Sturdy	CItr 13684	USA	null (Wx-A1b)	2	-	+	+	-	+	+
Fujimi Komugi	PI 360869	Japan	null (Wx-A1b)	2	-	+	+	-	+	+
Gabo	PI 155431	Australia	null (Wx-B1b)	3	+	-	+	+	-	+
Gamenya	PI 268329	Australia	null (Wx-B1b)	3	+	-	+	+	-	+
Santanta	CItr 14582	USA	null (Wx-B1b)	3	+	-	+	+	-	+
Bai Huo	PI 606717	China	null (Wx-D1b)	4	+	+	-	+	+	-
Kanto 107	PI 631445	Japan	null (Wx-A1b & B1b)	7	-	-	+	-	-	+
NSGC 8645	PI 612545	USA	Triple null alleles	8	-	-	-	-	-	-
NSGC 8646	PI 612546	USA	Triple null alleles	8	-	-	-	-	-	-

* Type classification based on combinations of three Wx null alleles [30]

^† ^+ = presence; - = absence.

### **Amplification of the *Wx *and *SSII *genes in diploid and tetraploid progenitors of hexaploid wheat**

Bread wheat (*Triticum aestivum *L.) is an allohexaploid (2n = 6x = 42) species with three (A, B and D) genomes. It originated from two natural hybridization events. The first one involved in the hybridization between *T. urartu *Thum. (A^u^A^u^) and an unknown species (BB) closely related to *Aegilops speltoides *Tausch. (SS), generating wild emmer *T. turgidum *ssp. *dicoccoides *(Körn. ex Aschers. & Graebn.) Thell. (AABB). The second event occurred between the domesticated emmer *T. turgidum *ssp. *dicoccon *Schrank (AABB) and *Ae. tauschii *Coss. (DD), resulting in the formation the hexaploid wheat *T. aestivum *L. (AABBDD) [33-35]. The first event happened less than half a million years ago, while the second event occurred only 8,000 years ago [33]. *T. monococcum L*. (A^m^A^m^), closely related to *T. urartu*, includes the wild einkorn, *T. monococcum *ssp. *aegilopoides *(syn. *T. boeoticum*) and the cultivated einkorn *T. monococcum *ssp. *monococcum*. *T. turgidum *ssp. *durum *Desf. is another domesticated form of *T. turgidum *ssp. *dicoccoides *[34,35].

**Figure 6:**
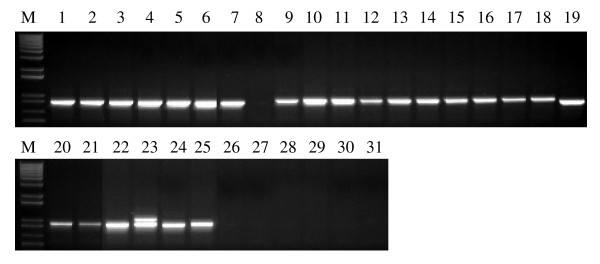
**PCR amplification of 18 diploid (*Triticum *and *Aegilops *species) and 13 tetraploid (wild and domesticated forms) progenitors of hexaploid wheat using the primer set SSII-7B-F2/SSII-7B-R2**. M = 1 kb plus DNA ladder. Lanes 1-2, *T. monococcum *accessions; lanes 3-5, *T. boeoticum *accessions; lanes 6-8, *T. urartu *accessions; lanes 9-13, *T. turgidum ssp. durum *accessions; lanes 14-18, *T. turgidum ssp. dicoccon *accessions; lanes 19-21, *T. turgidum ssp. dicoccoides *accessions; lanes 22-25, *Ae. speltoides *accessions; lanes 26-31, *Ae. tauschii *accessions.

These genome-specific primer sets for the three *Wx *and *SSII *genes were used to amplify 18 diploid (*Triticum *and *Aegilops *species) and 13 tetraploid (wild and domesticated forms) progenitors of hexaploid wheat (Additional file 1). It was found that the A genome specific primer sets designed from hexaploid wheat failed to yield any amplification products in most of the accessions of *T. monococcum *and *T. boeoticum *(the A^m ^genome), and *T. urartu *(the A^u ^genome), even though the A^u ^genome has been widely accepted as the A-genome donor of hexaploid wheat [33,34]. These results indicate significant sequence differences in the intron regions of *Wx *and *SSII *genes between the A^m ^(A^u^) genome and the A genome of bread wheat. The sequence differences could be insertion(s) and/or deletion(s). The most B genome-specific primer sets designed from hexaploid wheat could not generate amplification products in four accessions of *Ae. speltoides *(the S genome) (Additional file 1). The only primer set SSII-7B-F2/SSII-7B-R2 amplified two PCR fragments in one *Ae. speltoides *accession (PI 499261) and one fragment in other three *Ae. speltoides *accessions (Figure 6). These results also reveal sequence differences in the intron regions of *Wx *and *SSII *genes between the S genome and the B genome of bread wheat. Four D genome-specific primer sets for the *SSII-7D *genes could amplify PCR products with no length polymorphism from all six accessions of *Ae. tauschii*, the D genome donor of hexaploid wheat, whereas only one D genome-specific primer set Wx-7D-F2a/Wx-7D-R2a for the *Wx-7D *gene amplified a uniform fragment from six *Ae. tauschii *accessions, which was identical to that of hexaploid wheat (Additional file 1).

The identical PCR products amplified by A and B genome-specific primer sets for the *Wx *and *SSII *genes from hexaploid wheat were detected in 10 accessions of domesticated tetraploid wheat *T. turgidum *ssp. *dicoccon *and *T. turgidum *ssp. *durum*. This indicates that very high conservation of the *Wx *and *SSII *genes exists in the homologous A (B) genomes between cultivated durum and hexaploid wheat and these genome-specific primers can be used for the study of sequence diversity and association mapping of the three homoeologous *Wx *and *SSII *genes in natural populations of domesticated tetraploid wheat. However, no amplicon was observed in three accessions of wild tetraploid wheat *T. turgidum *ssp. *dicoccoides *(Additional file 1). These results indicate greater genetic variation in the *Wx *and *SSII *genes of the wild tetraploid wheat. It was interesting to find that PCR products were amplified in the accessions of *T. monococcum *and *T. boeoticum *(the A^m ^genome), and *T. urartu *(the A^u ^genome) by B genome specific primer sets (Wx-4A-F1/Wx-4A-R1 and SSII-7B-F2/SSII-7B-R2, Figure 6) and D genome-specific primer set SSII-7D-F3/SSII-7D-R3 (Additional file 1). This might be caused by mismatch PCRs between template DNA and primers [3].

Huang and Cloutier [4] reported that the same type LMW-GS genes are highly conserved in the homologous A (B) genomes between durum and hexaploid wheat, as well as in the homologous D genomes between hexaploid wheat and *Ae. tauschii*. Recent studies indicated that intergenic sequences in the *Glu-3 *regions are not conserved between the A^m ^genome of *T. monococcum *and the A genome of durum and bread wheats [36,37]. In the present study, we found very high conservation of *Wx *and *SSII *genes in the homologous A (B) genomes between durum and hexaploid wheats, as well as of *SSII-7D *gene between hexaploid wheat and *Ae. tauschii*. Large variations in the intron regions of *Wx *and *SSII *genes were observed in the wild diploid and tetraploid progenitors of hexaploid wheat. Therefore, in order to be able to compare genetic variation between the wild diploid/tetraploid progenitors and hexaploid wheat at the sequence level, primers can be developed from the conserved domains of exon regions after sequence alignment. The wild relatives of bread wheat containing novel alleles of *Wx *and *SSII *genes could be used as a valuable source for new genetic variation with the potential to improve wheat starch properties and starch contents.

## Conclusions

In summary, this paper describes a detailed and useful method for the development of genome-specific primers for the three homoeologous *Wx *and *SSII *genes in allohexaploid wheat. These genome-specific primers can be used for the study of sequence diversity and association mapping of the three homoeologous *Wx *and *SSII *genes in natural populations of both hexaploid wheat and cultivated tetraploid wheat. The strategies used in this paper can be used to develop genome-specific primers for agronomically important genes in any allopolypoid crops. They may be also suitable for (i) the development of gene-specific primers for duplicated paralogous genes in any diploid species, and (ii) the development of allele-specific primers at the same gene locus.

## Methods

### Plant materials and DNA isolation

Four nulli-tetrasomic (NT) lines (N4AT4D, N7AT7D, N7BT7D and N7DT7B) of 'Chinese Spring' (CS) were kindly provided by the Wheat Genetic and Genomic Resources Center (WGGRC), Department of Plant Pathology, Kansas State University, which were originally obtained from Dr. E.R. Sears [38]. These NT lines were used for chromosome assignment of PCR products.

Partial waxy wheat cultivars and breeding lines from different countries of the world (Table 2) were analyzed using the genome-specific primers for the *Wx *genes. These included the cultivars Fujimi Komugi and K107 from Japan; Bai-Huo and Chinese Spring from China; Gabo and Gamenya from Australia; NSGC 8645, NSGC 8646, Sturdy and Santanta from the United States. Seed of these lines was obtained from the Germplasm Resources Information Network (GRIN), USDA-ARS.

A total of 18 diploid *Triticum *and *Aegilops *species as well as 13 tetraploid wheat lines (*T. turgidum*, 2n = 4x = 14, AABB) were used to study the genetic variations of the *Wx *and *SSII *genes (Table 2). The diploid *Triticum *and *Aegilops *species included two *T. monococcum *and three *T. boeoticum *(2n = 2x = 14, A^m^A^m^), three *T. urartu *(2n = 2x = 14, A^u^A^u^), six *Ae. tauschii *(2n = 2x = 14, DD), and four *Ae. speltoides *(2n = 2x = 14, SS) accessions, whose genomes are closely related to A-, B-, and D-genome of the cultivated wheat (*T. aestivum*, 2n = 6x = 42, AABBDD), respectively [34]. These *Triticum *and *Aegilops *accessions were obtained from the Germplasm Resources Information Network (GRIN), USDA-ARS and the Plant Gene Resources of Canada (PGRC), Agriculture and Agri-Food Canada, respectively.

Total genomic DNA was extracted from young leaf tissue and frozen in liquid nitrogen, as previously described by Huang et al. [39]. The DNA was diluted to a concentration of 50 ng/μL before use for PCR.

### Primer design and PCR amplification

Genomic sequences of the three Wx (AB019622, AB019623 and AB019624) and three SSII genes (AB201445, AB201446 and AB201447), which were isolated by Murai et al. [13] and Shimbata et al. [15], respectively, were retrieved from the GenBank database http://www.ncbi.nlm.nih.gov/Genbank. Multiple alignment of nucleotide sequences were conducted by Bioedit 7.0 [40].

Primers were designed by eye based on the sequence polymorphism and general primer-picking parameters were checked using Primer3 http://frodo.wi.mit.edu/primer3/.

PCR reactions contained 200 ng template DNA, 1 × PCR buffer (Fermentas, 10 mM Tris-HCl pH 8.8, 50 mM KCl, and 0.08% Nonidet P40), 1.5 mM MgCl_2_, 0.2 mM of each dNTP, 0.6 μM of each primer, 1 U *Taq *DNA polymerase (recombinant, Fermentas) in a total volume of 25 μL. The PCR reaction was carried out in a MyCycler Thermo Cycler (Bio-Rad Laboratories Inc., Hercules, CA). After 5 min of initial denaturation at 94°C, 35 cycles were performed with 30 sec at 95°C, 30 sec at 60°C, 90 sec at 72°C and a final extension step of 10 min at 72°C. PCR products were separated on a 1% agarose gel in 0.5 × TBE buffer. The gel was photographed using a Molecular Imager Gel Doc XR system (Bio-Rad Laboratories Inc., Hercules, CA).

## Abbreviations

AGP: ADP-glucose pyrophosphorylase; BAC: bacterial artificial chromosome; CS: Chinese Spring; dNTP: deoxyribonucleotide triphosphate; GBSS: granule-bound starch synthase; Indel: insertion or deletion; LMW-GS: low molecular weight glutenin subunit; NT: nulli-tetrasomic; PCR: polymerase chain reaction; SBE: starch branching enzymes; SGP: starch granule protein; SNP: single nucleotide polymorphism; SS: starch synthases; TBE: tris borate EDTA; Tm: melting temperature; Wx: Waxy.

## Competing interests

The authors declare that they have no competing interests.

## Authors' contributions

XQH designed and carried out the experiments, collected materials, performed the sequence alignment, the primer design and the data analysis, and drafted the manuscript. ABB conceived the study and revised the manuscript. Both authors read and approved the final manuscript.

## Supplementary Material

Additional file 1 **Summary of PCR analysis in 31 diploid and tetraploid progenitors of wheat**. PCR analysis in 31 diploid and tetraploid progenitors of wheat using genome-specific primer sets for the Wx and SSII genes. Click here for file
